# Mr. Balfour on the Future of Our Race

**Published:** 1904-08-27

**Authors:** 


### The Hospital.

A Journal of

The Medical Sciences and Hospital Administration.

Vol. XXXVI.—No. 935. AUGUST 27, 1904.

### Mr. Balfour on the Future of Our Race.

The curiously pessimistic speech delivered by the Prime Minister in the anthropological section of the British Association, will hardly fail to excite very general attention, or to be received, we hope, with very general protest; while the vigorous antidote to it, which was furnished by Sir John Gorst, is certainly worthy of all commendation. Mr. Balfour referred in the first instance to the fine physical type furnished by the rural inhabitants of a Lowland district with which he was familiar in his youth, and in which a combination of large families with small cottages rendered a considerable degree of overcrowding inevitable, during the sleeping hours at least; and he founded upon this experience the suggestion that the real and essential distinction between the salubrity of town and country is to be sought in some absolute difference between their respective atmospheres, as if the town air in some manner or degree became, so to speak, devitalised before it reached the lungs of those whose position compelled them to breathe it. Such a condition, he suggested, would scarcely be improved by ventilation, because, if the air supplied were of inferior quality, its quantity would be a matter of only secondary importance. He passed on to consider some evidence brought before the section by Dr. Shrubsall, and tending to show that town life is favourable to the numerical preponderance of a dark-eyed and dark-haired race, the members of which are more liable than the light-eyed and fair-haired to pulmonary tuberculosis, to neuroses (including epilepsy), and to cancer. Dr. Shrubsall's paper, as reported, contained no reference to the locality in which his observations were conducted; and, admitting its conclusions to be correct for that locality, it by no means follows that they would be correct universally. Mr. Balfour, however, seemed to regard it as having "proved" the general existence of a change "of great importance," and, considering also that it would be the most energetic part of the rural population who drifted into towns and cities, he inferred that the burden of continuing the race would be largely thrown upon the less energetic who remained behind, and that deterioration must almost of necessity ensue. He concluded by saying that, after the Legislature had done what it could in respect of improved drainage, of improved ventilation, and of diminished overcrowding, he feared that the larger questions would not have been touched, that the essential difference between town and country life would still be left, and that no legislation was likely to modify the permanent causes by which not merely the well-being of this or the next generation was affected, but which concerned the actual quality of the race. We would fain hope that this view of the case is in great measure the outcome of the physical exhaustion consequent upon the labours of the Parliamentary session, and that the end of the recess may find Mr. Balfour in a more sanguine frame of mind. Such a note of despair, coming as it does from one to whom the country looks for leadership, cannot fail to cause some temporary consternation; and it is a considerable relief to know that an investigation of the premises on which Mr. Balfour based his remarks are scarcely sufficient to bear the weight of his conclusions.

In direct sequence to the Prime Minister's eminently unsatisfying pronouncement, Sir John Gorst stood up as the earnest and uncompromising advocate of a policy which would grapple boldly with all the manifest causes of disease or of degeneration, and would strive to bring them under control. Among those which he enumerated were premature marriages, the marriages of the mentally and physically unfit, and the frequent lack of the fresh air and food which were so essential to the proper development of the young. He glanced also at the recent report of the Royal Commission which has investigated the causes of the alarming decrease in the birth-rate in Australia, and has attributed it largely to the refusal by the middle classes, on account of growing luxury and selfishness, to perform their duty and to bear the burden of bearing and bringing up children for the State. Such a condition, wherever existing, must of necessity tend towards an undue preponderance of the offspring of the comparatively unfit. Sir John went on to say that the causes which actively injured child-life from birth to manhood in this country were operative not only in the homes, but in the schools for which society and the Government are directly responsible; and he strongly urged that power should be given to local authorities for dealing experimentally with conditions of this kind which might be conspicuous in the districts under their control. He pointed out the absolute necessity of feeding before teaching, and the want of means of enforcing upon parents the necessity of feeding their children properly. Charity might mitigate the existing evils, but in doing so it produced the very neglect by parents which it should be a chief object to prevent. To force a hungry or continually underfed child to any exertion of mind or body was absolute cruelty, such that it would be followed by prosecution if it were practised upon a horse. There were plenty of methods for feeding children and for coercing parents into the discharge of their parental duties, and some of these means were already adopted upon the Continent. If we add to Sir John Gorst's recommendations the further and important one that the Local Government Board should be empowered to deal promptly and effectively with the neglects and omissions of local sanitary authorities, and to enforce compliance with existing laws upon the subject of nuisances and other conditions tending towards the production or the diffusion of disease, we feel sure that a very notable amelioration in the condition of our urban populations might in no long time be effected. The proposed continuous anthropometric survey will almost certainly be carried into effect, and it should be the business, alike of the central government and of local authorities, so to order themselves that the completion of this survey may afford ample encouragement to renewed endeavour.

